# Safety of Influenza A H1N1pdm09 Vaccines: An Overview of Systematic Reviews

**DOI:** 10.3389/fimmu.2021.740048

**Published:** 2021-10-28

**Authors:** Lene Kristine Juvet, Anna Hayman Robertson, Ida Laake, Siri Mjaaland, Lill Trogstad

**Affiliations:** Division of Infection Control and Environmental Health, Norwegian Institute of Public Health, Oslo, Norway

**Keywords:** H1N1pdm09 vaccination, pandemic vaccines, influenza vaccines, safety, adverse events

## Abstract

**Background:**

In 2009, a new influenza A H1N1 virus emerged causing a global pandemic. A range of monovalent influenza A H1N1pdm09 vaccines with or without adjuvants were developed. After the mass vaccination campaigns safety concerns related to H1N1pdm09 vaccines were reported. More than a decade later, reported AEFIs are still under scrutiny. We performed a systematic review aiming to synthesize the evidence on the safety of the H1N1pdm09 vaccines on reported outcomes from existing systematic reviews.

**Methods:**

Four electronic databases, PubMed, EMBASE, Epistimonikos and the Cochrane Database of Systematic Reviews were searched for articles on H1N1pdm09 vaccination published from 2009 to January 2021. Systematic reviews assessing short- or long-term adverse events after H1N1pdm09 vaccination were considered for inclusion. Data was extracted from all selected reviews. Outcomes were grouped and results from each included review were presented narratively and in tables.

**Results:**

16 systematic reviews met the inclusion criteria. Reported outcomes were short-term events (3 reviews), fetal/pregnancy outcomes (8 reviews), Guillain-Barré syndrome (GBS) (4 reviews), narcolepsy (2 reviews) demyelinating diseases (1 review based on one study only) and inflammatory bowel disease (IBD) (1 review). Short-term serious adverse events were rare, 3 cases amongst 16725 subjects in 18 randomized controlled trials (0.018%). No deaths were reported. The risks of local events were generally higher for adjuvanted vaccines as compared to unadjuvanted vaccines. Maternal H1N1pdm09 vaccination in any trimester was not associated with an increase in preterm birth, small for gestational age, congenital malformations or fetal death. For GBS, results were conflicting. The main systematic review on narcolepsy found a 5-14-fold increased risk in children, and a 2-7- fold increased risk in adults after vaccination with Pandemrix. The attributable risk of narcolepsy one year after vaccination was 1 case per 18 400 vaccine doses in children/adolescents, and 1 case per 181 000 vaccine doses in adults.

**Conclusion:**

Adjuvanted vaccines had more local but not serious adverse events compared to unadjuvanted vaccines. Vaccination with Pandemrix was strongly associated with narcolepsy, particularly in children. No increased risks of pregnancy outcomes were seen after pandemic vaccination. The findings on GBS were inconclusive.

## Introduction

In 2009, a novel H1N1 influenza A virus (H1N1pdm09) emerged causing a global pandemic. According to Centers for Disease Control and Prevention (CDC) an estimated 151,700 - 575,400 people worldwide died from H1N1pdm09 virus infection during the first year the virus circulated (1). Globally, 80 percent of H1N1pdm09 virus-related deaths were estimated to have occurred in people younger than 65 years of age. This differs greatly from typical seasonal influenza epidemics, during which about 70 to 90 percent of deaths are estimated to occur in people 65 years and older (2). Pregnant women were early considered to be at increased risk of severe disease and adverse fetal outcomes (3).

To combat the pandemic virus, a range of monovalent H1N1pdm09 vaccines were developed, mainly drawing on existing egg-based technology from seasonal influenza vaccines. The vaccines were produced with the adjuvants AS03, MF59, aluminium, or without adjuvants. By June 2010, more than 350 million people had received H1N1pdm09 vaccines worldwide (4). In Europe, more than 37 million people were vaccinated with three centrally authorized Influenza A H1N1 vaccines marketed in the European Economic Area: Celvapan (no adjuvants), Focetria (MF59 adjuvanted) and Pandemrix (AS03 adjuvanted). More than 30 million persons received Pandemrix in Europe (4, 5). The overall effectiveness of the pandemic vaccines has been estimated to 80% (95% CI 59-90%) against laboratory confirmed influenza, with adjuvanted vaccines being significantly more effective in children than adults (6). Pandemic vaccination may also have contributed to less severe outcomes related to H1N1pdm09 infection in the following flu season (2010/11) when the same virus strain continued to circulate (7).

The safety of vaccines is a prime concern, also in pandemic situations. Safety monitoring systems require coordinated actions and collaboration between regulatory and immunization program authorities on a national level and concerted international efforts to maintain proper management and public trust. In response to the pandemic influenza A H1N1pdm09 strain, mass vaccination campaigns administrating vaccines to large populations over a short period of time were launched. In such situations, surveillance and evaluation of adverse events following immunization (AEFIs) may be particularly challenging due to large numbers of vaccine adverse events reports. An AEFI is defined as any untoward medical occurrence which follows immunization and which does not necessarily have a causal relationship with the vaccine (8). The fact that a vaccine was administered within a reasonable time period of the occurrence of an event does not automatically suggest that the vaccine caused or contributed to the event. Nevertheless, a temporal association is necessary to imply causation. In many countries, the vaccination campaigns coincided with the pandemic peak. This may have complicated the evaluation of suspected AEFIs, which in some cases may be difficult to separate from symptoms or consequences of the pandemic influenza infection itself, for instance Guillain-Barré Syndrome (GBS) or Chronic fatigue syndrome/Myalgic encephalopathy (CFS/ME) (9, 10). The evidence of a link between a vaccine as a potential cause and a specific event is derived from well- designed population based epidemiological studies (8). Since clinical trials are not powered to detect *rare* adverse events, large, prospective studies including appropriate comparison groups are crucial. Knowledge on the expected background rates of possible adverse events is important for the assessment of possible vaccine adverse reactions. Other health conditions may occur in close proximity to vaccination in a substantial number of people when large populations are vaccinated. Thus, careful evaluation of vaccine safety signals is critical to detect the true vaccine reactions and to establish whether coincidental events were caused by vaccination or not.

A number of reports on suspected AEFIs have been published, among which the unexpected increased incidence of narcolepsy in children and young adults following vaccination with Pandemrix received massive attention among the general public and medical communities, in particular in Europe. A number of observational studies have confirmed the association between Pandemrix vaccination and narcolepsy (11–14), whereas studies on associations between H1N1pdm09 vaccination and other outcomes have shown no or conflicting results (9, 10, 15). More than a decade later, reported AEFIs after H1N1pdm09 vaccination are still under scrutiny and assessment for causality in Norway, and a synthesis of the available evidence warranted. The objective of this systematic review was to synthesize the current evidence on the safety of the H1N1pdm09 vaccines from existing systematic reviews, based on both randomized controlled trials and observational studies.

## Methods

### Search Strategy and Selection of Systematic Reviews

The search strategy followed the Preferred Reporting Items for Systematic Review and Meta-analysis (PRISMA) guidelines (16, 17). Four electronic databases, namely, PubMed, EMBASE, Epistimonikos and the Cochrane Database of Systematic Reviews, were searched. Keywords employed were (“H1N1 pdm09” OR “influenza pandemic 2009”) AND (“vaccin*” OR “pandemic vaccine*”) (**Supplementary Table 1**). The search was designed to identify primary studies and systematic reviews, and covered literature published between 2009 and November 2019. All retrieved studies were imported into the Rayyan QCRI (18) and duplicated articles were removed.

Criteria for inclusion were short- or long-term adverse events after H1N1pdm09 vaccination compared to a control group (**Table 1**). Two independent researchers initially screened all articles based on title and abstract, categorizing them as “included”, “excluded” or “maybe”. Any disagreements or “maybes” were resolved by consensus with a third reviewer. For this study, only systematic reviews were included. Systematic reviews limited to vaccine efficacy were excluded. Only publications in English were included. An updated search for systematic reviews in Pubmed (systematic review filter) was performed on 22^nd^ January 2021.The reference lists were checked for further systematic reviews not previously identified. Subsequently, full text assessment of the included systematic reviews was performed by two reviewers to determine study eligibility based on the inclusion and exclusion criteria. Disagreements were resolved by discussion including a third reviewer.

**Table 1:** Review inclusion criteria (PICO).

**Population**	All children, women and men.
**Intervention**	Pandemic vaccine during season 2009-2010.
**Comparisons**	No vaccination, placebo or other vaccines
**Outcome**	**Safety – outcomes all** • Acute events • Local adverse events • Longterm events • Systemic adverse events **Safety – additional outcomes pregnant women** • Spontaneous abortion, foetal death, stillbirth, preterm birth (less than 37 weeks), pre-eclampsia and eclampsia • Neonatal outcomes: congenital malformations (minor and major), neonatal death.
**Study designs**	Systematic reviews, health technology assessments

### Assessment of Methodological Quality of Included Reviews

Two reviewers independently assessed the quality of each review using the revised “A Measurement Tool to Assess systematic Reviews, version 2” (AMSTAR 2) (19). Disagreements were resolved by discussion and, if necessary, arbitration among the whole review team. The level of confidence in the findings of the reviews was assessed according to the number of critical and minor flaws in the methodology. Only two systematic reviews included a list of excluded studies (Q7) (20, 21). If the systematic review included a flow chart explaining the reason for exclusion, it was scored as partial yes (PY). The source of funding (Q10) for the incorporated observational studies was not reported in the systematic reviews and was categorized as not applicable (NA). For most reviews, too few studies were included to enable assessment of publication bias. If the authors justified why the assessment could not be performed, the item was scored ‘yes’ (Q15).

### Data Extraction and Management

One reviewer extracted data from all selected reviews into a spreadsheet (Microsoft Excel) including number and settings of the included trials, total number and characteristics of participants, intervention(s) assessed, outcomes measured and major limitations. A second reviewer cross-checked the extracted data for accuracy. Extracted variables from each systematic review are presented in detail in the characteristics of included systematic reviews (**Table 2**). Only data on H1N1pdm09 vaccines were extracted.

**Table 2:** AMSTAR2 rating of 16 included systematic reviews.

Systematic review	AMSTAR2 rating																Confidence in findings of review
	Q1	Q2	Q3	Q4	Q5	Q6	Q7	Q8	Q9	Q10	Q11	Q12	Q13	Q14	Q15	Q16	
**Demicheli 2018** (20)	Y	Y	Y	Y	Y	Y	Y	Y	Y	Y	Y	N	N	Y	Y	Y	High
**Fell 2015** (22)	Y	Y	Y	Y	Y	Y	PY	Y	Y	NA	NA	NA	Y	Y	Y	Y	High
**Foo 2020** (23)	Y	Y	Y	Y	Y	Y	PY	Y	Y	NA	NA	NA	Y	Y	NA	Y	High
**Giles 2019** (24)	Y	N	Y	PY	Y	Y	PY	Y	Y	NA	N	N	N	Y	Y	Y	Moderate
**Hauser 2019** (25)	Y	Y	Y	Y	Y	Y	PY	Y	Y	Y	Y	N	N	Y	Y	Y	High
**Manzoli 2011** (26)	Y	N	Y	Y	Y	Y	PY	Y	Y	Y	N	Y	Y	N	Y	Y	Moderate
**Martin Arias 2015** (27)	Y	N	Y	Y	Y	Y	PY	PY	N	NA	Y	N	N	Y	Y	N	Moderate
**McMillan 2015** (21)	Y	Y	Y	Y	Y	Y	Y	Y	Y	Y	Y	Y	Y	Y	N	Y	High
**Nunes 2016** (28)	Y	N	Y	Y	Y	Y	PY	Y	N	NA	Y	N	N	Y	Y	Y	Moderate
**Pineton 2015** (29)	Y	N	Y	Y	Y	N	PY	Y	N	NA	NA	NA	N	Y	NA	Y	Moderate
**Polyzos 2015** (30)	Y	N	Y	Y	Y	Y	PY	Y	Y	NA	Y	N	N	Y	Y	Y	Moderate
**Sanz Fadrique 2019** (31)	Y	N	Y	PY	N	N	PY	PY	N	NA	NA	NA	N	N	NA	Y	Low
**Sarkanan 2018** (32)	Y	N	Y	Y	Y	Y	PY	Y	N	NA	Y	N	Y	Y	Y	Y	Moderate
**Stassijns 2016** (33)	Y	N	Y	Y	N	N	N	Y	N	N	Y	N	N	Y	N	Y	Low
**Wachira 2019** (34)	Y	PY	Y	PY	Y	Y	PY	Y	Y	NA	NA	NA	Y	N	NA	N	Moderate
**Zhang 2018** (35)	Y	N	Y	Y	Y	Y	PY	Y	Y	NA	N	N	N	N	NA	Y	Moderate

1. components of PICO, 2. established protocol prior to review, 3. selection of study design, 4.comprehensive literature search, 5. study selection in duplicate, 6.data extraction in duplicate, 7. list of excluded studies, 8.describe the included studies, 9. assessing the risk of bias, 10. sources of funding, 11.meta-analysis if appropriate, 12. meta-analysis sensitivity RoB, 13. interpreting RoB when discussing the results, 14. discussing heterogeneity, 15. investigation publication bias, 16. potential sources of conflict of interest.

N, no; NA, not applicable no meta-analysis conducted; PY, partial yes; Y, yes.

## Results

**Figure 1:**
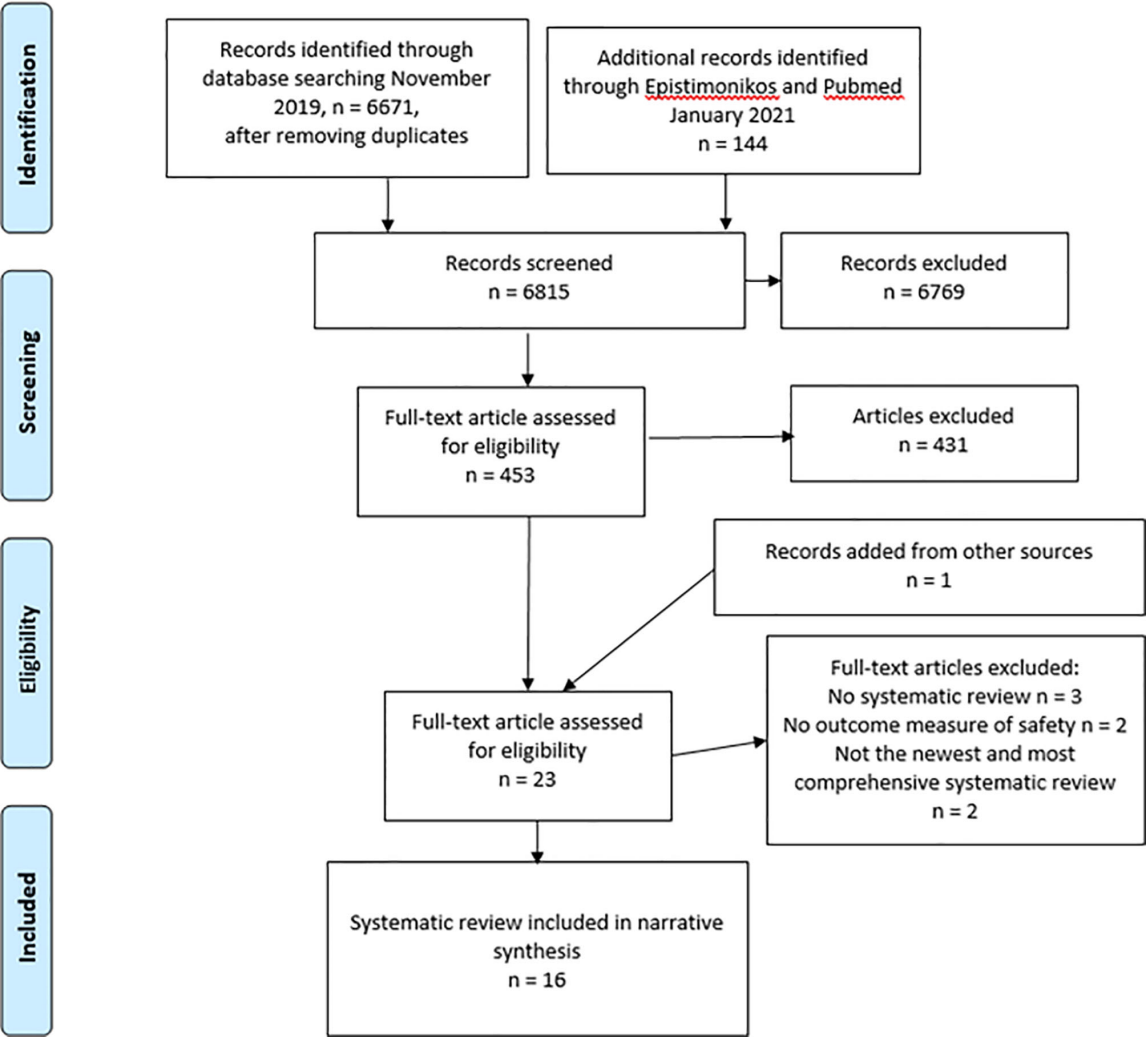
Study selection, PRISMA flow chart.

After exclusion of duplicates, the initial literature search identified 6815 articles for abstract review (**Figure 1**). After excluding articles based on abstract review, 453 remained. In the current study only systematic reviews were included (**Table 1**), and 22 systematic reviews were selected for full-text review according to the inclusion criteria. One additional article was found through hand searching of other literature. Of the 23 systematic reviews, 7 were excluded (**Supplementary Table 2**), and 16 reviews were included in the overview.

**Table 2** summarizes the quality assessments of the included reviews. Most of the reviews were of moderate or high quality, but for two reviews there was low confidence in the findings of the review (**Table 2**). Common critical domain deficiencies included failure to preregister the review protocol (Q2), and failure to list excluded studies (Q7). Failure to consider risk of bias when interpreting results (Q13) were also quite frequent.

**Table 3** shows an overview of the outcomes covered by the included systematic reviews. The main outcomes were short-term adverse events, narcolepsy, Guillain-Barré syndrome (GBS) and pregnancy- or fetal outcomes. One systematic review also included a single study on inflammatory bowel disease (IBD) (29), and one systematic review included a single study on demyelinating disease (20). Most studies reporting short-term adverse events were randomized controlled trials (RCTs), whereas reviews on rare or long-term outcomes were based on observational studies.

**Table 3:** Overview of included systematic reviews on H1N1pdm09 vaccines according to outcome.

OUTCOME	Reference	Total number of studies included^a^	Type of study	Meta-analyses by vaccine type (adjuvants yes/no)	Date of search
**Short term adverse events (mild/mod/serious)**					
All types	Manzoli, (26)	18	RCT	Yes	Apr. 2011
Effect of adjuvants, pediatric (all types)	Stassijns, ^b^ (33)	8	RCT	Yes	Apr. 2015
Effect of adjuvants, pediatric/adults (mild only)	Hauser, (25)	22^c^	RCT	Yes	Sep. 2018
**Narcolepsy**	Sarkanen, (32)	11	Observational studies	Yes	Nov. 2016
	Demicheli, (20)	4		No	Dec. 2016
**Guillain-Barré syndrome (GBS)**	Sanz Fadrique, ^d^ (31)	2	Observational studies	No	Jul. 2017
	Martin Aries, (27)	16		Yes	Apr. 2014
	Demicheli, (20)	2		No	Dec. 2016
	Wachira, (34)	15		No	Jun. 2017
**Inflammatory bowel disease (IBD)**	Pineton, (29)	1	Observational study	NA	Jun. 2014
**Demyelinating diseases**	Demicheli, (20)	1	Observational study	NA	Dec. 2016
**Pregnancy- and fetal outcomes and offspring**					
Adverse events in pregnancy (local, systemic, preeclampsia); congenital malformation; spontaneous abortion; still birth; preterm birth; small for gestational age (SGA); low birth weight	McMillan, (21)	17	Observational studies	No	Mar. 2014
Preterm birth; late fetal death; any fetal death	Fell, (22)	12	Observational studies	No	May 2013
Preterm birth; SGA; low birth weight	Nunes, (28)	13	Observational studies	No	Jun. 2015
Congenital malformation	Polyzos, (30)	12	Observational studies	No	Dec. 2014
Spontaneous abortion; fetal death; stillbirth; preterm birth; congenital malformations; neonatal death	Demicheli, (20)	14	Observational studies	No	Dec. 2016
Congenital malformation; spontaneous abortion; still birth; preterm birth; SGA	Zhang, (35)	19	Observational studies	No	Jan. 2017
Congenital malformation; stillbirth/fetal death; SGA; low birth weight	Giles, (24)	9	Observational studies	Yes	May 2017
**Early childhood health outcomes**	Foo, (23)	6	Observational studies	No	Jul. 2019

a studies from which data extraction on H1N1pdm09 vaccination was possible (i.e. not pooled with seasonal influenza vaccination). In systematic reviews covering more than one outcome, the number of studies may be lower for single outcomes. ^b^studies also included in Hauser et al. (25). ^c^unclear if all studies reported adverse events. ^d^update of Martin Aries et al. (27).

### Short-Term Adverse Events

Three systematic reviews on short-term adverse events were included (25, 26, 33). These reviews included only RCTs. Manzoli addressed all types of adverse events and all types of vaccines (26). Hauser (25) assessed the effects of adjuvants on mild adverse events, whereas Stassijsn (33) was limited to the effect of adjuvants on mild to serious adverse events in pediatric populations only. In general, direct meta-analysis comparing rates of adverse events for different vaccines, dosing regimens and adjuvants was challenging due to varying definitions of adverse events and types of events reported, and events frequently being reported as percentages only. According to Manzoli (26), the proportion of serious adverse events was low (0.018%, 3 cases amongst 16725 subjects, 18 RCTs), and no deaths were reported. They found a minor dose effect on local and systemic events for non-adjuvanted vaccines (based on 6 studies where one dose was used, and 6-7 studies where two doses were used), whereas this was not found for adjuvanted vaccines (one study), but data was scarce.

Aluminium containing vaccines were associated with an increased risk of local events compared to unadjuvanted vaccines (26) (**Supplementary Table 3**). In adults, the risk of local adverse events after vaccination with oil-in water adjuvant containing vaccines (AS03 or MF59) was higher compared to vaccination with unadjuvanted vaccines (25, 26). The increased risk was significantly higher for AS03 adjuvanted vaccines compared to MF59 adjuvanted vaccines, RR = 2.90 (95% CI 2.37-3.54) for AS03 and RR = 1.70 (95% CI 1.25-2.31) for MF59, subgroup difference p< 0.004 (25). No difference in risk associated with adjuvants was observed for systemic events (fever). The data was more limited in children. In Stassijns (33), 29 trials encompassing more than 25 000 children were included, but only four trials included information on AS03, and four trials included information on MF59. No overall increase in serious adverse events was seen in the AS03 trials nor in MF59 trials for children, and no overall increase in solicited or unsolicited AEFIs was found (33). Local pain was reported with rates between 31.7-84.6% for AS03 adjuvanted vaccines, and 1.0-59% for MF59 adjuvanted vaccines (33). Hauser reported a possible increase in local adverse events with MF59 adjuvanted vaccines in children but had no information on AS03 adjuvanted pandemic vaccine. The Hauser review (25) was assessed to high quality, the Manzoli review (26) to moderate and the Stassijns review (33) to low quality (**Table 2**).

### Narcolepsy

Two systematic reviews on narcolepsy was included. The systematic review by Sarkanen included 29 studies (32). Only 11 studies, all on Pandemrix, were included in the meta-analysis. The analyses were performed separately for children/adolescents summarized from nine studies: Finland, France, Ireland, the Netherlands, Norway, Sweden and the UK. For adults, the meta-analysisadults included 5 studies: Finland, France, Ireland, Sweden and the UK. The analysis was specified for three different proxy dates for onset of disease (index dates). The studies included 376 narcolepsy cases and 5.1 million subjects/person years in vaccinated children/adolescents; 95 narcolepsy cases and 11.3 million subjects/person years in unvaccinated children/adolescents; 133 narcolepsy cases and 9.0 million subjects/person years in vaccinated adults; and 59 narcolepsy cases and 12.1 million subjects/person years in unvaccinated adults. Increased risk of narcolepsy type 1 after vaccination with Pandemrix was found in children/adolescents for all index dates. In the meta-analysis the overall RRs were 14.3 (95% CI 8.9-23.0), 9.7 (95% CI 4.9- 19.2), and 5.0 (95% CI 3.4-7.5) for onset of symptoms, first healthcare contact, and diagnosis, respectively (**Table 4**). Based on studies included in the meta-analysis, the attributable risk one year after vaccination was 1 case per 18 400 vaccine doses (95% CI 1 per 16 700 to 1 per 20 400) in children/adolescents (based on 5 studies) and 1 case per 181 000 vaccine doses (95% CI 1 per 141 000 to 1 per 254 000) in adults (based on 3 studies). Increased risk of narcolepsy type 1 was also observed in adults, although the association was not as strong as in children/adolescents, overall RRs were 7.0 (95% CI 3.4-14.5), 8.1 (95% CI 3.9-16.9), and 3.0 (95% CI 1.9-4.62) for onset of symptoms, first healthcare contact, and diagnosis, respectively. The heterogeneity between studies was generally very low.

**Table 4:** Systematic review of vaccination with Pandemrix and risk of narcolepsy.

	Children and adolescents			Adults		
	Number of studies	Effect size (95% CI)	I^2^	Number of studies	Effect size (95% CI)	I^2^
**Index date**^**a**^						
Onset date	6	14.32 (8.92, 22.99)	0.0%	3	7.01 (3.40, 14.46)	0.0%
Healthcare contact	3	9.68 (4.88, 19.23)	44.1%	3	8.08 (3.86, 16.89)	0.0%
Diagnosis	5	5.02 (3.36, 7.51)	0.0%	4	2.95 (1.88, 4.62)	0.0%

a Exact date of symptom onset is difficult to remember and prone to recall bias. The studies used different index dates as proxy of disease onset. Some studies are included in analyses of more than one index date.

Narcolepsy incidence was not increased in countries where other H1N1pdm09 vaccines than Pandemrix were used: South Korea, US and Canada (Ontario). In Quebec, Canada, where AS03-adjuvanted Arepanrix vaccine was used, RR 16 weeks after vaccination was 1.48 -4.32 based on different study designs. The vaccine attributable risk was only 1 per 1,000,000, which is significantly lower than in European studies. In a qualitative synthesis of 12 studies, the authors did not find evidence of increased risk of narcolepsy after vaccination with non-Pandemrix H1N1pdm09 vaccines, including Arepanrix (AS03-adjuvanted) and MF59 adjuvanted vaccines. The authors also reported some evidence of rising incidence of narcolepsy in relation to H1N1pdm09 infection, referring to studies from the Beijing and Shanghai area with a 3-fold increase in narcolepsy incidence 3-6 months after the pandemic peak in a largely unvaccinated population. The Sarkanen review (32) was assessed to moderate quality (**Table 2**).

The Cochrane systematic review by Demicheli (20) only provided a brief description of 4 studies (2 of which had overlapping datasets) on narcolepsy following pandemic vaccination, together with other neurological and autoimmune diseases, confirming the increased risk in children. These studies were from Finland, France and Ireland and were also covered by Sarkanan (32). The Cochrane review was assessed to high quality (**Table 2**).

### Guillain-Barré Syndrome (GBS)

GBS is an acute autoimmune disorder which attacks the nervous system. A meta-analysis from 2015 (27), an updated review from 2019 (31), a meta-analysis from 2018 (20) and a narrative systematic review from 2019 (34) were included. 16 studies were incorporated in the meta-analysis by Martin, Arias (27) and an overall RR = 1.84 (95% CI 1.36-2.50) of GBS after pandemic vaccination was estimated. However, heterogeneity was high (I^2^ = 64%) and only 7 of the 16 studies found a significantly increased risk. A funnel plot did not identify publication bias. Risk estimates were higher in meta-analysis based on self-controlled analyses compared to other study designs. The risk estimates of GBS after vaccination varied according to geographic region, although not significantly, estimates being higher in Australia and Taiwan (RR = 3.54, 95% CI 1.05-11.97), and lower in Europe (RR = 1.62, 95%CI 0.83-3.13). Estimates for adjuvanted vaccines and unadjuvanted vaccines compared to unvaccinated were similar RR = 1.97 (95%CI 1.22–3.17) and RR = 1.75 (95%CI 1.20–2.56). The estimates were based on 7 and 9 studies respectively. The updated review (31) only identified two new studies, one from South Korea which found a significant association (RR = 1.46, 95% CI 1.26-1.68), and a registry study from Norway which found no association after adjustment for influenza infection (HR = 1.1, 95% CI 0.51-2.43) (**Supplementary Table 4**). No updated meta-analysis was performed.

A newer systematic review by Wachira (34) identified 15 articles of which only two found a statistically significant association between H1N1pdm09 vaccines and GBS. Crude estimates from 10 primary studies were presented in a Forrest plot without a pooled estimate. There was a significant association (RR = 2.8 95% CI 1.3-6.0) in one of the studies, but according to the authors, this association disappeared when adjusted for influenza like illness, infections of the respiratory tract and other seasonal influenza vaccines (RR = 1.0. CI 95% 0.3-2.7). Wachira (34) only covered five of the studies included in the analysis of Martin Arias, thus 11 studies were not covered despite similar inclusion criteria with regards to study design (**Supplementary Table 4**).

The Cochrane review by Demicheli (20) from 2018 included two case control studies on H1N1pdm09 vaccination and GBS in general populations in a meta-analysis (**Supplementary Table 4**). In the crude analyses, the odds of GBS after vaccination was two-fold increased. However, the odds ratio was reduced after adjustment for pandemic influenza infection, other diseases and medication, indicating no increased risk (OR 0.92 (0.35-2.4). The studies of Martin Arias and Wachira were both assessed to moderate quality according to the AMSTAR-2 tool, while the Cochrane review by Demicheli (20) was assessed to high quality (**Table 2**).

### Inflammatory Bowel Disease (IBD)

One systematic review that assessed risk of IBD after vaccination was included (29). Only one study on H1N1pdm09 vaccine (Pandemrix) was included in the review. Overall, people vaccinated with H1N1pdm09 vaccine did not have significantly higher risk of IBD compared to the unvaccinated, HR = 1.13 (95% CI 0.97-1.32). The Pineton review (29) was assessed to moderate quality (**Table 2**).

### Demyelinating Diseases

One review (20) (high quality, **Table 2**) assessed the association between H1N1pdm09 vaccination and risk of demyelinating diseases. The review included only one study, and the presented OR was unadjusted, OR = 2.06 (95% CI 0.51-8.22). The study was conducted in individuals vaccinated with the MF59-adjuvanted H1N1pdm09 vaccine Focetria.

### Fetal Outcomes

Seven systematic reviews on fetal outcomes, all based on observational studies were included (**Table 4**) (20–22, 24, 28, 30, 35). Three of the systematic reviews were assessed to high quality (20–22), while the others were assessed to moderate quality (24, 28, 30, 35) (**Table 2**). The reviews provided evidence on the outcomes congenital malformations, spontaneous abortion, stillbirth/fetal death, preterm birth, small for gestational age birth (SGA), and low birth weight (LBW). Not all systematic reviews included all outcomes. A list of included primary studies for each outcome is provided in **Supplementary Table 5**. All the systematic reviews compared vaccinated/exposed individuals to unvaccinated/unexposed individuals. Some of the systematic reviews included studies with both H1N1pdm09 monovalent vaccine and seasonal vaccines, but only the results from studies with H1N1pdm09 vaccines (both adjuvanted and non-adjuvanted) were included here.

Estimates from five systematic reviews on congenital malformations were all close to one (**Table 5**) (20, 21, 24, 30, 35). Only one review found a significant association (OR = 1.14 (95% CI 1.01-1.29) (35), while three other reviews found no significant association with vaccination (OR = 1.02 (95% CI 0.91-1.17) (30), (OR = 1.11 (95% CI 0.99- 1.29) (20) and OR = 1.03 (95% CI 0.99, 1.07) (24). The last review also suggested no association (no pooled estimate) (21). Only two primary studies were included in all five systematic reviews (**Supplementary Table 5**).

**Table 5:** Adjusted estimates for fetal outcomes after maternal H1N1pdm09 vaccination.

Outcome/ Systematic review	Vaccine adminstrated	Congenital malformations			Spontaneous abortion			Stillbirth/Fetal death/Abortion			Preterm delivery (< 37 weeks)			Small for gestational age birth (SGA)			Low birth weight (LBW)		
		Studies	Effect size (95% CI)	I^2^	Studies	Effect size (95% CI)	I^2^	Studies	Effect size (95% CI)	I^2^	Studies	Effect size (95% CI)	I^2^	Studies	Effect size (95% CI)	I^2^	Studies	Effect size (95% CI)	I^2^
**Fell** (22)	*Any trimester*							3	Range 0.56-0.79^a^		10	No association^a^							
									Range 0.89-1.23^ab^										
									Range 0.44-0.77^ac^										
**McMillan (** 21 **)**	*Any trimester*	7	No association^a^		5	No association^a^		9	No pooled estimate		6	OR = 0.93 (0.83-1.04)	59%	2	OR = 0.91 (0.87-0.96)	0%	6	OR = 0.94 (0.82-1.08)	39%
											3	HR = 1.00 (0.93-1.07)^d^	0%						
	*Any trimester*										2	OR = 0.79 (0.61-1.01)^e^	19%						
**Polyzos (** 30 **)**	*Any trimester*	10	OR = 1.02																
			(0.91-1.14)																
	*First trimester*	6	OR = 1.02																
			(0.89-1.17)																
**Nunes (** 28 **)**	*Any trimester*										9	OR = 0.90 (0.82-0.99)	72%	6	OR = 0.98 (0.91-1.07)	54%	7	OR = 0.88 (0.79-0.98)	62%
**Zhang (** 35 **)**	*Any trimester*	6	OR = 1.14	0%	3	OR = 1.04	0%	10	OR = 0.80	8%	12	RR = 0.92	68%	7	OR = 0.98	45%			
			(1.01-1.29)			(0.72-1.52)			(0.69-0.92)			(0.84-1.01)			(0.91-1.06)				
	*First trimester*	2	OR = 1.07	0%															
			(0.59-1.94)																
**Demicheli (** 20 **)**	*Any trimester*	6	OR = 1.11	0%				5	OR = 0.75	0%	7	OR = 0.84	71% 59%						
			(0.99-1.23)					3	(0.62-0.90)^f^		2	(0.76-0.93)							
									HR = 0.81			HR = 1.11^d^							
									(0.63-1.04)^df^	0%		(0.46-2.68)							
	*Second/third trimester*										2	OR = 1.08	0%						
												(0.92-1.28)							
	*First trimester*										2	OR = 0.96	0%						
												(0.87-1.90)							
**Giles (** 24 **)**	*Any trimester*	7	OR = 1.03 (0.99-1.07)	0%				3	OR = 0.84	0%									
									(0.65-1.08)										
	*Second/third trimester*	1	HR = 0.96 (0.29-3.12)								3	OR = 0.96 (0.87-1.06)	0%	3	OR = 0.96 (0.89-1.04)	0%	2	OR = 0.97 (0.71-1.32)	83%
	*First trimester*	1	HR = 1.32 (0.78-2.21)								2	OR = 1.08 (0.92-1.28)	0%				2	OR = 1.00 (0.80-1.24)	0%

a no pooled estimate, and no I^2 b^late fetal death ^c^early fetal death ^d^separate analysis on time metric and calculated HR ^e <^32 weeks ^f^abortion included spontaneous, internal, foetal death and stillbirth.

Two reviews explored the relationship between maternal H1N1pdm09 vaccination and spontaneous abortion (21, 35) (**Table 5**). Neither review found any association between maternal H1N1pdm09 vaccination (any trimester) and spontaneous abortion. Only Zhang et al. presented a pooled estimate [OR 1.04 (95% CI 0.72-1.52)] for spontaneous abortion prior to gestational week 22 (35).

Five systematic reviews on stillbirth/fetal death/abortion were included (20–22, 24, 35) (**Table 5**). Two concluded there was no evidence of increased risk of preterm birth after H1N1pdm09 vaccination, but the studies were too heterogeneous to be pooled (21, 22). Three other systematic reviews performed meta-analyses (20, 24, 35). All found effect estimates below one, consistent with no increased risk of fetal death following maternal H1N1pdm09 vaccination.

The six systematic reviews on preterm birth found no evidence that maternal H1N1pdm09 vaccination was associated with an increase in preterm birth in any trimester (20–22, 24, 28, 35) (**Table 5**). In five of the reviews, estimates for vaccination in any trimester were below one. In three of these, confidence intervals included one (21, 24, 35). One review did not perform a meta-analysis (22). One review included an estimate for very preterm birth (21) (**Table 5**).

There was consistent evidence of no increased risk of SGA after maternal H1N1pdm09 vaccination in any trimester, reported in four systematic reviews (21, 24, 28, 35) (**Table 5**). Three reviews with meta-analyses found no association (24, 28, 35). The last review suggested a very small protective effect for the vaccine on SGA birth when pooling two studies (21).

Three reviews evaluated the relationship between H1N1pdm09 vaccination and LBW (21, 24, 28) (**Table 5**). There was no association in meta-analyses that included studies of vaccination in any trimester (21), in the second and third trimester combined (24), or in the first trimester (24). One review observed a lower rate of LBW after maternal H1N1pdm09 vaccination, although the confidence interval was wide (28).

One review (24) did a separate analysis for adjuvanted H1N1pdm09 vaccines. The estimates for SGA, LBW, preterm birth and congenital abnormalities were all around 1 with confidence intervals that included 1. These estimates were similar to the estimates combining both adjuvanted and unadjuvanted vaccines.

Only the Cochrane review addressed neonatal death (20). The review was based on two studies and suggested that pandemic vaccine during pregnancy was not associated with an increased risk of neonatal death OR = 1.09 (95% CI 0.4-2.95).

In a narrative systematic review based on five cohort studies, no significant association was found between pandemic vaccination and preeclampsia (21).

### Long Term Effects in Children Following Maternal H1N1pdm09 Vaccination

A narrative systematic review by Foo et al. (23) was the only review concerning long-term effects of H1N1pdm09 vaccination during pregnancy on early childhood health outcomes. The review identified six primary studies which assessed the effect on influenza infections, primary infections only, childhood mortality up to the age of 5, and two registry studies assessing the effect on infections, hospitalisations, and general diseases and syndromes. No association between maternal vaccination and adverse health outcomes in early childhood were identified. The review was assessed to high quality (**Table 2**).

## Discussion

Overall, 16 systematic reviews on adverse events following vaccination with monovalent H1N1pdm09 vaccines were included. According to the AMSTAR 2 assessment tool, five of the systematic reviews were considered high quality (20–23, 25), two were considered low quality (31, 33). The rest were considered moderate quality.

Overall, the risk of short term serious adverse events was low following H1N1pdm09 vaccination. In clinical trials, adjuvanted vaccines had more local, but not more serious adverse events compared to unadjuvanted vaccines. Vaccination with Pandemrix was strongly associated with narcolepsy, particularly in children. For GBS, the findings from the systematic reviews were inconsistent. Two other outcomes identified in the systematic reviews were IBD (29) and demyelinating diseases (20). For these outcomes, the estimates were based on only one primary study, thus no conclusions could be drawn.

Almost half of the systematic reviews covered fetal outcomes after maternal vaccination (20–22, 24, 28, 30, 35), and in general indicated no increased risk of adverse pregnancy outcomes. Furthermore, studies did not reveal any adverse effect of maternal H1N1pdm09 vaccination on childhood health outcome during the first 5 years of life (23).

### Adverse Events by Vaccine and Adjuvants

All included reviews based on RCTs performed meta-analyses according to vaccine type/adjuvants. Among reviews based on observational studies, only three performed meta-analyses according to vaccine type/adjuvants (24, 27, 32). However, several of the reviews included tables of included primary studies with information on vaccine type (24, 30, 35). For rare events like GBS or adverse pregnancy outcomes (fetal death, SGA, LBW, premature birth or spontaneous abortion), no differences were reported according to adjuvanted or non-adjuvanted pandemic vaccines, or type of adjuvant (MF59, AS03) in any of the included reviews. Increased risk of narcolepsy was only seen following vaccination with the AS03-adjuvanted vaccine Pandemrix, however not for the AS03-adjuvanted vaccine Arepanrix, as discussed below.

### Narcolepsy

Although the absolute numbers of children and young adults developing narcolepsy type 1 were limited to around 400 reported cases across the included studies. In Europe, H1N1pdm09 vaccination with Pandemrix was consistently associated with an increased risk of narcolepsy (32). During the first year after vaccination, the relative risk of narcolepsy was increased 5 to 14-fold in children and adolescents and 2 to 7-fold in adults. The vaccine attributable risk in children and adolescents was around 1 per 18,400 vaccine doses and 1 per 181, 000 in adults. The risk was limited to vaccination with the Pandemrix vaccine only and was only found for narcolepsy type 1. Follow-up time in the included studies was up to approximately two years, and onset of symptoms occurred most often during the first three to six months following vaccination. The Cochrane systematic review by Demicheli (20) only provided information from studies also covered by Sarkanan (32). Narcolepsy incidence was not increased in countries where other H1N1pdm09 vaccines than Pandemrix were used: South Korea, US and Canada (Ontario). In Quebec, Canada, where AS03-adjuvanted Arepanrix vaccine was used, the vaccine attributable risk was only 1 per 1,000,000, which is significantly lower and not comparable to the large excess risks demonstrated in European studies. According to the authors, it cannot completely be ruled out that this finding may be due to a confounding effect of H1N1pdm09 influenza infection (36).

Increased incidence of narcolepsy in absence of pandemic vaccination was reported from Beijing and Shanghai following the pandemic peak (37, 38). The incidence decreased back to baseline two years after the H1N1 pandemic, suggesting that infection with the 2009 H1N1 strain was associated with narcolepsy onset. In many countries, the vaccination campaigns coincided with the pandemic peak, thus dual exposure to pandemic influenza infection and vaccine was likely. Also, in Germany the incidence of narcolepsy increased threefold starting in spring 2009, although the overall pandemic vaccine coverage was only 4-8%. Thus, a role also for natural H1N1pdm09 infection in the development of narcolepsy is possible. Moreover, a combined effect of simultaneous exposure to H1N1pdm09 infection and vaccination on the risk of narcolepsy cannot be ruled out, since mass vaccination campaigns coincided with the pandemic peak in some countries (13). Confounding by natural H1N1pdm09 infection was briefly discussed by the authors of the systematic review. Increased risk of narcolepsy was only seen following vaccination with the AS03-adjuvanted vaccine Pandemrix. However, no clear increased risk was reported after vaccination with the AS03 adjuvanted vaccine, Arepanrix, which was made by the same vaccine producer, but at another production facility (32, 39). This observation lends support to the recent hypothesis of molecular mimicry of a specific configuration of the vaccine antigen (40) as a potential causal factor in the development of narcolepsy, rather than the AS03 adjuvant (41).

### Guillain-Barré Syndrome (GBS)

One of the systematic reviews found a significant association between H1N1pdm09 vaccination and GBS (27) based on a pooled estimate of 16 studies, whereas another systematic review (34) found few primary studies supporting this finding. There was little overlap between the primary studies included, despite similar inclusion and exclusion criteria in terms of study design (cohort, case control, self-controlled case series and self- controlled risk interval design). However, the objective of Wachira’s review was broader, and aimed at discovering any aetiological agents of GBS, and the searches were carried out in different databases (34). In contrast, Demicheli (20) only included two case control studies on GBS. The inclusion criteria were narrow and did not include self-controlled case series, which are commonly used for very rare outcomes, such as GBS. Demicheli (20) assessed the two studies as unclear risk of bias, whereas Wachira (34) gave the same studies a high rating, both according to the Newcastle Ottowa quality assessment Scale. The cohort studies included in Wachira (34) also gained high ratings, though the case series received somewhat lower ratings. These discrepancies illustrate how authors may emphasize certain factors over others when performing systematic reviews.

Wachira (34) explored all known infectious aetiological agents of GBS, reconfirming *Campylobacter jejuni* as one of the main triggers of GBS, in addition to other infections including influenza like illness (6/7 studies). Importantly, one study showed a strong association with H1N1pdm09 infection (HR = 4.22 95%CI 1.01-17.59) in contrast to pandemic vaccination in the same population, where no association was found (9). The review by Demicheli (20) found a two-fold increased risk of GBS in crude analyses. However, similar to the findings of Wachira (34), the odds ratio was reduced after adjustment for pandemic influenza infection, indicating no increased risk. As the pandemic peak and vaccination campaign coincided in many countries, exposure to both influenza infection and vaccine was likely (13). Also, the epidemiology of gastrointestinal infections like *Campylobacter jejuni* may depend on population and setting, explaining the geographical differences in estimates (27). However, obtaining good data on infection is generally challenging for most study populations/settings and a difficult confounder to control for. Thus, lack of control for coincident infections might to some extent explain the lack of consistency in studies on influenza vaccines and risk of GBS, although other factors cannot be ruled out. Given that the systematic reviews on GBS had different approaches and inconsistent results, novel analysis would be beneficial for this outcome.

### Pregnancy Outcomes

In general, no associations with H1N1pdm09 vaccination were found for any of the fetal outcomes assessed. Three of the seven reviews were considered high quality (20–22). Only one review performed sub-analysis according to adjuvated vaccine *versus* no vaccine (24) and did not find any difference in the risk of adverse pregnancy outcome. Early in the pandemic, pregnant women were identified as at high risk of serious complications (3). The WHO therefore recommended that pregnant women regardless of pregnancy length received the vaccine, and policies were widely adopted after 2009 pandemic (42). Consequently, there was an immediate need for knowledge on the safety of pandemic vaccines, especially on fetal outcomes, and these initial studies also formed part of evidence base for the safety of seasonal influenza vaccination. Nearly all the primary studies were conducted in high-income countries, and less is known on safety of maternal H1N1pdm09 vaccination in low- and middle-income countries. Small inconsistencies between the reviews were observed and may be attributable to the difference in inclusion of primary studies (**Supplementary Table 5**). The primary studies included in the reviews may also differ in terms of study design, baseline immunity to influenza, coincidence between vaccine and pandemic influenza season, or not considering immortal time bias. The systematic review on long-term effects of maternal H1N1pdm09 vaccination found no association between maternal vaccination and adverse health outcomes in early childhood (23). The authors of the systematic review concluded that this field is under-investigated.

### Strengths and Limitations

Overviews of systematic reviews relating to the adverse effects of an intervention may allow commonalities to be drawn across a broader range of evidence than in a more focused systematic review, with the potential to highlight equivalence or patterns not previously identified (43). The suitability of reanalysis of existing data within an overview is debated. It has been argued that, where novel analyses are the aim, conducting a review of primary studies may be more appropriate than an overview of reviews (43). Using existing results of literature searches may nevertheless save time (44).

Even though systematic reviews increasingly try to consider all outcomes (both beneficial and harmful), data on adverse events may be more fragmented and incomplete, and given more cursory treatment than efficacy/effectiveness data. The decision to perform meta-analysis on included studies can differ between systematic reviews (45), due to different approaches often described as ‘lumping’ or ‘splitting’ of information. Lumping refers to finding commonalities across different approaches, whereas splitting creates a more narrowly refined focus within a broader research field (43). Such decisions require both sufficient knowledge of the subject area, both for exposures and outcomes, which often represent different specialities, as well as competence in the methodology of systematic reviews and meta-analysis. This was apparent both for GBS and for the pregnancy outcomes, whereby the systematic reviews seemed to provide different justifications for or against meta-analysis (for e.g. degree of heterogeneity).

A limitation of our systematic review is that we may not have identified all the systematic reviews covering safety outcomes in our search result. This may especially be true for reviews including studies that are primarily designed to address vaccine efficacy/effectiveness, with additional short- term safety data.

### Future Challenges

Mass vaccination against the H1N1pdm09 pandemic illustrated that rare, unexpected adverse events can occur, which are almost impossible to predict. Clinical trials are not powered to assess rare or long-term events due to the urgent need for prevention. In practice, rare and/or long-term events will therefore not be detected until mass vaccination is carried out through post-marketing surveillance and well-designed observational studies with comparison groups are conducted. Furthermore, as was the case during the 2009 pandemic, H1N1pdm09 virus circulation and vaccination coincided, and hence it is difficult to disentangle the effects of infection from vaccination, or indeed the effect of dual exposure (9, 13, 38, 44). In hindsight, the H1N1 2009 pandemic was less severe than anticipated, and subsequently led to an adaptation of the WHO pandemic phases to ensure disease severity was incorporated in the pandemic criteria – in addition to incidence of disease (46). In contrast, the current SARS-CoV-2 pandemic has been associated with a significantly higher disease burden and the risk willingness for vaccination may be higher. This will likely affect vaccine uptake.

### Relevance in Current and Future Pandemics/Epidemics

In the event of new pandemics, caused by influenza or other agents, novel vaccines will be developed. In a pandemic situation with new vaccines it will be impossible to foresee new serious adverse events. Careful evaluation of the short- and long-term effects of both the infection itself, as well as the vaccine used for prevention, should be performed. This is highly actualized in the COVID-19 pandemic where long-term consequences of COVID-19 infection is becoming evident (47) and mass vaccination campaigns with vaccines based on new technologies have been rolled out (48, 49) where case reports on serious hematological adverse events have been published for difference vaccines (50–52).

In terms of surveillance and epidemiological studies on safety of pandemic vaccination there are lessons to be learnt from the 2009 H1N1 pandemic. Causality assessment of AEFIs should firstly be performed at the population level, to establish if there is a causal association between the use of a vaccine and a particular AEFI in the population. In the evaluation of individual AEFI case reports, population-based evidence should be reviewed, and a logical deduction performed to determine whether an AEFI in a specific individual is causally related to the use of the vaccine (8). Furthermore, ensuring sufficient data-collection on all relevant outcomes and exposures including both pandemic infection and vaccination, with appropriate control groups is crucial.

## Conclusion

Twelve years after the 2009 H1N1 pandemic, adverse events following administration of the H1N1pdm09 vaccines have been rigorously studied. Adjuvanted vaccines had more local, but not serious, adverse events compared to unadjuvanted vaccines. Vaccination with Pandemrix was consistently associated with narcolepsy, particularly in children. Although Pandemrix was an adjuvanted vaccine, molecular mimicry of a specific configuration of the vaccine antigen has been suggested as a potential causal factor in the development of narcolepsy, rather than the AS03 adjuvant. Pregnant women were at increased risk of severe influenza illness and adverse pregnancy outcomes, however there is no evidence of adverse effects in mothers nor children following H1N1pdm09 vaccination in pregnancy. The findings on GBS were inconclusive. In conclusion, the risk benefit of the H1N1pdm09 vaccines appear favorable.

## Data Availability Statement

The original contributions presented in the study are included in the article/**Supplementary Material**. Further inquiries can be directed to the corresponding author.

## Author Contributions

All the authors contributed with selection and assessment of the included systematic reviews, in addition to writing and completed the manuscript. All authors contributed to the article and approved the submitted version.

## Funding

The Norwegian Institute of Public Health funded this study.

## Conflict of Interest

The authors declare that the research was conducted in the absence of any commercial or financial relationships that could be construed as a potential conflict of interest.

## Publisher’s Note

All claims expressed in this article are solely those of the authors and do not necessarily represent those of their affiliated organizations, or those of the publisher, the editors and the reviewers. Any product that may be evaluated in this article, or claim that may be made by its manufacturer, is not guaranteed or endorsed by the publisher.
