# Supplementary material for: Safety of Influenza A H1N1pdm09 Vaccines: An Overview of Systematic Reviews
Source: Front Immunol. 2021 Oct 28;12:740048. doi: 10.3389/fimmu.2021.740048 (PMC8581668; doi:10.3389/fimmu.2021.740048)
Supplement: Supplementary file 1 [file Table_1.docx]

**Supplementary table 1. Search strategies**

**Databases: Ovid MEDLINE(R) and Epub Ahead of Print, In-Process & Other Non-Indexed Citations, Daily and Versions(R) <1946 to November 19, 2019>**

**Date:** 20.11.2019

**Hits:** 4686

| 1 | Influenza vaccines/ | 22108 |
| --- | --- | --- |
| 2 | Influenza A Virus, H1N1 Subtype/ | 14902 |
| 3 | ("A/H1N1pdm09" or swineflu or "swine flu" or "A/H1N1/09" or "pH1N1*" or (pandemic adj1 (influenza or flu))).tw,kf. | 9391 |
| 4 | 2 or 3 | 19713 |
| 5 | 1 and 4 | 5104 |
| 6 | (pandemic adj5 (vaccin* or immuni?ation)).tw,kf. | 2508 |
| 7 | (Pandemrix or Arepanrix or Celvapan or Focetria or Panenza or Panvax or Cantgrip or Fluval or Celtura or Humenza or "2009 Monovalent").tw,kf. | 294 |
| 8 | 5 or 6 or 7 | 6090 |
| 9 | Animal/ not (animal/ and human/) | 4612990 |
| 10 | 8 not 9 | 5465 |
| 11 | limit 10 to yr="2009 -Current" | 4686 |

**Database: Embase 1974 to 2019 February 27**

**Date**: 21.11.2019

**Hits**: 3866

| 1 | Influenza vaccine/ | 35518 |
| --- | --- | --- |
| 2 | 2009 H1N1 influenza/ | 3686 |
| 3 | ("A/H1N1pdm09" or swineflu or "swine flu" or "A/H1N1/09" or "pH1N1*" or (pandemic adj1 (influenza or flu))).tw,kw. | 11549 |
| 4 | 2 or 3 | 13545 |
| 5 | 1 and 4 | 4060 |
| 6 | (pandemic adj5 (vaccin* or immuni?ation)).tw,kw. | 3179 |
| 7 | (Pandemrix or Arepanrix or Celvapan or Focetria or Panenza or Panvax or Cantgrip or Fluval or Celtura or Humenza or "2009 Monovalent").tw,kw. | 988 |
| 8 | 5 or 6 or 7 | 5858 |
| 9 | (animal/ or exp nonhuman/ or Animal experiment/) not ((animal/ or exp nonhuman/ or Animal experiment/) and exp human/) | 5866672 |
| 10 | 8 not 9 | 5239 |
| 11 | limit 10 to ((conference abstracts or embase) and yr="2009 -Current") | 3866 |

**Database**: **Cinahl**

**Date**: 21.11.2019

**Hits**: 1074

| S9 | S5 OR S6 OR S7 Limiters - Published Date: 20090101-20191231 | 1,074 |
| --- | --- | --- |
| S8 | S5 OR S6 OR S7 | 1,248 |
| S7 | TI(Pandemrix or Arepanrix or Celvapan or Focetria or Panenza or Panvax or Cantgrip or Fluval or Celtura or Humenza or "2009 Monovalent") or AB(Pandemrix or Arepanrix or Celvapan or Focetria or Panenza or Panvax or Cantgrip or Fluval or Celtura or Humenza or "2009 Monovalent") | 78 |
| S6 | TI(pandemic N4 (vaccin* or immuni#ation)) or AB(pandemic N4 (vaccin* or immuni#ation)) | 534 |
| S5 | S1 AND S4 | 927 |
| S4 | S2 OR S3 | 4,186 |
| S3 | TI("A/H1N1pdm09" or swineflu or "swine flu" or "A/H1N1/09" or "pH1N1*" or (pandemic N0 (influenza or flu))) or AB("A/H1N1pdm09" or swineflu or "swine flu" or "A/H1N1/09" or "pH1N1*" or (pandemic N0 (influenza or flu))) | 3,230 |
| S2 | (MH "Influenza, Pandemic (H1N1) 2009") | 1,602 |
| S1 | (MH "Influenza Vaccine") | 9,439 |

**Database:** **PsycINFO <1806 to November Week 2 2019>**

**Date:** 22.11.2019

**Hits:**  159

| 1 | Immunization/ | 4439 |
| --- | --- | --- |
| 2 | Swine influenza/ | 172 |
| 3 | ("A/H1N1pdm09" or swineflu or "swine flu" or "A/H1N1/09" or "pH1N1*" or (pandemic adj1 (influenza or flu))).tw. | 451 |
| 4 | 2 or 3 | 538 |
| 5 | 1 and 4 | 126 |
| 6 | (pandemic adj5 (vaccin* or immuni?ation)).tw. | 98 |
| 7 | (Pandemrix or Arepanrix or Celvapan or Focetria or Panenza or Panvax or Cantgrip or Fluval or Celtura or Humenza or "2009 Monovalent").tw. | 18 |
| 8 | 5 or 6 or 7 | 178 |
| 9 | (animal not (animal and human)).po. | 355277 |
| 10 | 8 not 9 | 177 |
| 11 | limit 10 to yr="2009 -Current" | 159 |

**Database:** **CRD - The Database of Abstracts of Reviews of Effects (DARE), NHS Economic Evaluation Database (NHS EED) og HTA**

**Date:** 22.11.2019

**Hits:**  43 (removed hits before 2009)

| 1 | MeSH DESCRIPTOR Influenza vaccines IN DARE,NHSEED,HTA | 201 |
| --- | --- | --- |
| 2 | MeSH DESCRIPTOR Influenza A Virus, H1N1 Subtype IN DARE,NHSEED,HTA | 56 |
| 3 | (("A/H1N1pdm09" or swineflu or "swine flu" or "A/H1N1/09" or "pH1N1*" or (pandemic NEAR1 (influenza or flu)) or ((influenza or flu) NEAR1 pandemic))) IN DARE, NHSEED, HTA | 74 |
| 4 | #2 OR #3 | 104 |
| 5 | #1 AND #4 | 34 |
| 6 | ((pandemic NEAR4 (vaccin* or immuni?ation))) IN DARE, NHSEED, HTA | 23 |
| 7 | ((Pandemrix or Arepanrix or Celvapan or Focetria or Panenza or Panvax or Cantgrip or Fluval or Celtura or Humenza or "2009 Monovalent")) IN DARE, NHSEED, HTA | 1 |
| 8 | #5 OR #6 OR #7 | 43 |
