# Supplementary material for: Safety of Influenza A H1N1pdm09 Vaccines: An Overview of Systematic Reviews
Source: Front Immunol. 2021 Oct 28;12:740048. doi: 10.3389/fimmu.2021.740048 (PMC8581668; doi:10.3389/fimmu.2021.740048)
Supplement: Supplementary file 2 [file Table_2.docx]

**Supplementary tables 2. List of excluded systematic reviews**

| Systematic review | Reasons for exclusion |
| --- | --- |
| Cardenas, G., Soto-Hernandez, J. L., Diaz-Alba, A., Ugalde, Y., Merida-Puga, J., Rosetti, M., & Sciutto, E. (2014). Neurological events related to influenza A (H1N1) pdm09. Influenza & Other Respiratory Viruses, 8(3), 339-346. | This is not a systematic review |
| Halsey, N. A., Griffioen, M., Dreskin, S. C., Dekker, C. L., Wood, R., Sharma, D., . . . Kelso, J. M. (2013). Immediate hypersensitivity reactions following monovalent 2009 pandemic influenza A (H1N1) vaccines: reports to VAERS. Vaccine, 31(51), 6107-6112. | Including VAERS reports and not studies with a control group |
| Lansbury, L. E., Smith, S., Beyer, W., Karamehic, E., Pasic-Juhas, E., Sikira, H., . . . Nguyen-Van-Tam, J. S. (2017). Effectiveness of 2009 pandemic influenza A(H1N1) vaccines: A systematic review and meta-analysis. Vaccine, 35(16), 1996-2006. | The systematic review does not include safety outcomes |
| Leite, A., Andrews, N. J., & Thomas, S. L. (2016). Near real-time vaccine safety surveillance using electronic health records-a systematic review of the application of statistical methods. Pharmacoepidemiology and Drug Safety, 25(3), 225-237. | This systematic review does not include safety outcomes |
| Manzoli, L., Ioannidis, J. P., Flacco, M. E., De Vito, C., & Villari, P. (2012). Effectiveness and harms of seasonal and pandemic influenza vaccines in children, adults and elderly: a critical review and re-analysis of 15 meta-analyses. Human vaccines & Immunotherapeutics, 8(7), 851-862. | This is an overview of systematic overviews and not a systematic review, and refers to the systematic reviews by Manzoli 2011 (included) and Yin 2011 (excluded) |
| McMillan, M., Porritt, K., Kralik, D., Costi, L., & Marshall, H. (2015). Influenza vaccination during pregnancy: a systematic review of fetal death, spontaneous abortion, and congenital malformation safety outcomes. Vaccine, 33(18), 2108-2117. | Same systematic review as one of the included comprehensive systematic review by McMillan [21], but this is the article version. |
| Yin, J. K., Khandaker, G., Rashid, H., Heron, L., Ridda, I., & Booy, R. (2011). Immunogenicity and safety of pandemic influenza A (H1N1) 2009 vaccine: systematic review and meta-analysis. Influenza & Other Respiratory Viruses, 5(5), 299-305. | Not the newest systematic review of short-term safety outcome |
