# Supplementary material for: Safety of Influenza A H1N1pdm09 Vaccines: An Overview of Systematic Reviews
Source: Front Immunol. 2021 Oct 28;12:740048. doi: 10.3389/fimmu.2021.740048 (PMC8581668; doi:10.3389/fimmu.2021.740048)
Supplement: Supplementary file 5 [file Table_5.docx]

**Supplementary Table 5. Primary studies included in systematic reviews on H1N1pdm09 vaccination and pregnancy outcomes.**

| **Primary studies/ SR** | **McMillan 2014** | **Polyzos 2015** | **Zhang 2018** | **Giles 2019** | **Demicheli 2018** |
| --- | --- | --- | --- | --- | --- |
| Mackenzie 2011 |  | x |  |  |  |
| Heikkinen 2012 | x | x | x | x | x |
| Kallen 2012 | x | x |  | x | x |
| Lin 2012 |  | x |  |  |  |
| Oppermann 2012 | x | x | x | x | x |
| Pasternak 2012 | x | x | x |  | x |
| Chambers 2013 | x | x | x |  |  |
| Clearly 2014 |  | x |  |  |  |
| Rubenstein 2013 | x | x |  |  |  |
| Louik 2013 | x | x |  |  |  |
| Trotta 2013,2014 |  | x | x | x | x |
| Fabiani 2015 |  |  | x |  |  |
| Ludvigsson 2015 |  |  |  | x |  |
| Launay 2012 |  | x |  |  | x |

1. **Congenital malformations**

| **Primary studies/ SR** | **Fell 2014** | **McMillan 2014** | **Nunes 2016** | **Zhang 2018** | **Giles 2019** | **Demicheli 2018** |
| --- | --- | --- | --- | --- | --- | --- |
| Fell 2012 | x | x | (x) | x |  | x |
| Heikkinen 2012 | x |  | x | x | x | x |
| Kallen 2012 | x | x | x | x | x | x |
| Lin 2012 |  | x |  |  |  |  |
| Ludvigsson 2013 | x |  | x | x | x | x |
| Oppermann 2012 | x |  |  |  |  |  |
| Pasternak 2012 | x | x | x | x | x | x |
| Pasternak 2012 |  | x | (x) |  |  |  |
| Sammon 2012 | x |  |  |  |  |  |
| Chambers 2013 |  |  | (x) | x |  |  |
| Clearly 2014 |  |  | x |  |  | x |
| Håberg 2013 | x | x | x |  |  |  |
| Richards 2013 | x | x | (x) | x |  | x |
| Rubenstein 2013 | x |  | x | x |  | x |
| Louik 2013 |  | x | (x) | x |  | x |
| Beau 2014 | x | x | x | x |  | x |
| Baum 2015 |  |  |  | x |  |  |
| Fabiani 2015 |  |  | x | x |  |  |
| Van der Maas 2015 |  |  | (x) |  | x |  |
| Cantu 2013 |  | x | (x) |  |  |  |

**b. Preterm delivery/birth**

1. **Fetal death / stillbirth /abortion**

| **Primary studies/ SR** | **Fell 2014** | **McMillan 2014** | **Zhang 2018** | **Giles 2019** | **Demicheli 2009** |
| --- | --- | --- | --- | --- | --- |
| Fell 2012 | x | x | x |  | x |
| Heikkinen 2012 | x | x | x | x | x |
| Kallen 2012 | x | x |  | x | x |
| Launay 2012 |  |  |  |  | x |
| Opperman 2012 |  |  |  |  | x |
| Pasternak 2012 | x | x | x |  | x |
| Sammon 2012 | x | x |  |  |  |
| Chambers 2013 |  | x |  |  |  |
| Håberg 2013 | x | x | x |  | x |
| Rubenstein 2013 |  | x | x |  |  |
| Beau 2014 | x |  | x |  | x |
| Pasternak 2014 |  |  | x |  |  |
| Trotta 2013,2014 |  |  | x | x |  |
| Baum 2015 |  |  | x |  |  |
| Ludvigsson 2015 |  |  | x |  |  |
| Cantu 2013 |  | x |  |  |  |

1. **Small for gestational age birth**

| **Primary studies/ SR** | **McMillan 2014** | **Nunes 2016** | **Zhang 2018** | **Giles 2019** |
| --- | --- | --- | --- | --- |
| Fell 2012 | x | (x) |  |  |
| Kallen 2012 |  | x | x |  |
| Ludvigsson 2013 |  | x | x | x |
| Pasternak 2012 | x | x |  | x |
| Chambers 2013 |  | (x) | x |  |
| Richards 2013 | x | x | x |  |
| Beau 2014 |  | x | x |  |
| Pasternak 2014 |  |  | x |  |
| Trotta 2014 |  | x | x |  |
| Van der Maas 2015 |  | (x) |  | x |
| Cantu 2013 | x | (x) |  |  |

1. **Low birth weight**

| **Primary studies/ SR** | **McMillan 2014** | **Nunes 2016** | **Giles 2019** |
| --- | --- | --- | --- |
| Heikkinen 2012 |  | x |  |
| Kallen 2012 | x | x |  |
| Lin 2012 | x |  |  |
| Ludvigsson 2013 |  | x | x |
| Pasternak 2012 | x | x | x |
| Pasternak 2012 | x |  |  |
| Håberg 2013 |  | x |  |
| Richards 2013 | x |  |  |
| Rubenstein 2013 |  | x |  |
| Fabiani 2015 |  | x |  |
| Cantu | x |  |  |
